# Non-alcoholic fatty liver disease severity is modulated by transglutaminase type 2

**DOI:** 10.1038/s41419-018-0292-8

**Published:** 2018-02-15

**Authors:** Mauro Piacentini, Andrea Baiocchini, Franca Del Nonno, Gerry Melino, Nickolai A. Barlev, Federica Rossin, Manuela D’Eletto, Laura Falasca

**Affiliations:** 1 National Institute for Infectious Diseases, IRCCS “Lazzaro Spallanzani”, Rome, Italy; 2 Department of Biology, University of Rome “Tor Vergata”, Rome, Italy; 3 Department Experimental Medicine and Surgery, University of Rome “Tor Vergata”, Rome, Italy; 4 Institute of Cytology Russian Academy of Sciences, Saint-Petersburg, Russian Federation

## Abstract

Non-alcoholic fatty liver disease (NAFLD) is one of the most important liver diseases worldwide. Currently, no effective treatment is available, and NAFLD pathogenesis is incompletely understood. Transglutaminase type 2 (TG2) is a ubiquitous enzyme whose dysregulation is implicated in the pathogenesis of various human diseases. Here we examined the impact of TG2 on NAFLD progression using the high-fat-diet-induced model in both wild-type and TG2-deficient mice. Animals were fed with a standard chow diet or a high-fat diet (42% of the energy from fat) for 16 weeks. Results demonstrated that the absence of a functional enzyme, which causes the impairment of autophagy/mitophagy, leads to worsening of disease progression. Data were confirmed by pharmacological inhibition of TG2 in WT animals. In addition, the analysis of human liver samples from NAFLD patients validated the enzyme’s involvement in the liver fat disease pathogenesis. Our findings strongly suggest that TG2 activation may offer protection in the context of NAFLD, thus representing a novel therapeutic target for tackling the NAFLD progression.

## Introduction

Non-alcoholic fatty liver disease (NAFLD) is a pathological change characterized by the accumulation of fat, called steatosis, which is found at least in 5% of hepatocytes. NAFLD is an increasingly recognized condition that has become the most common liver disorder in developed countries, with prevalence estimates around 24% in Europe^1^. It is closely associated with features of the metabolic syndrome such as obesity, insulin-resistance, type 2 diabetes, and hyperlipidemia^2^. In patients with chronic hepatitis C, steatosis has a prevalence of 40–86% and its frequency varies with genotype; it is more common in genotype 3 infection, where it occurs in 73% of patients, while the prevalence of steatosis in patients infected with other genotypes is around 50%^3^.

NAFLD is a spectrum of disorders, beginning as simple steatosis which can evolve into non-alcoholic steatohepatitis (NASH) and fibrosis, often resulting in cirrhosis and even hepatocellular carcinoma^4^.

The clinical importance of NAFLD and the current lack of effective medications to limit or reverse disease progression in patients with NASH have aroused great interest and intense investigation into the basic mechanisms involved in the disease’s development and progression. Hepatic fat accumulation results from an imbalance between triglycerides acquisition and removal^5^. Classically, the NAFLD physiopathology and progression has been summarized in the “two hits” hypothesis, with the first hit being steatosis, and the oxidative stress being involved in the second hit leading to the progression to NASH^6^. A multiple-hit hypothesis is now recognized, in which the timing and combination of genetic, external, and intracellular events, rather than the simple sequence of hepatic insults, result in different pathways, which lead to steatosis or NASH, respectively^7^. The enzyme transglutaminase type 2 (TG2) is a part of the cell response evoked by stress conditions, and its deregulation has been demonstrated to be involved in inflammatory and fibrotic diseases^8^. TG2 is a ubiquitous member of the TG family. Under pathological conditions it can be located in the extracellular matrix (ECM) or at the cell surface in association with the ECM^9^ as well as in the cytoplasm, where it is mostly soluble; it is also associated, however, with the inner face of the plasma or nuclear membrane^10^, and in the mitochondria^11^. TG2 has been implicated in a variety of cellular processes, such as differentiation, cell death, inflammation, cell migration, and wound healing^12–14^. In addition, we have demonstrated that TG2 is an essential component for the proper maturation of autophagosomes under basal and particularly under stressful cellular conditions^15^.

Considering all these findings we explored whether TG2 could be involved in the development of fatty liver disease. To this end the pathogenesis of NAFLD was analyzed in vivo using a TG2-null mouse model exposed to an experimental nutritional induction. Data obtained showed that TG2 deficiency is a key factor to limit NAFLD progression.

## Results

### Changes in body and liver weight

**Fig. 1:**
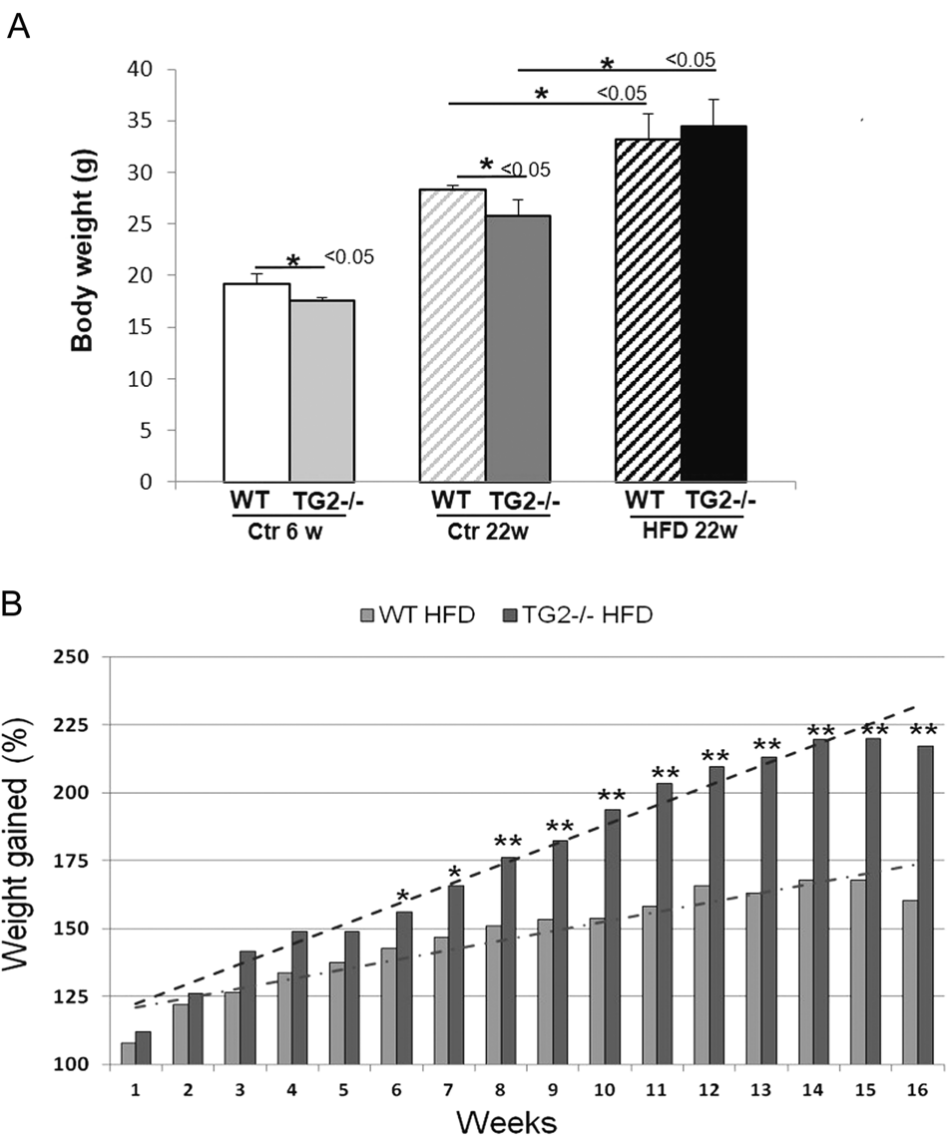
Longitudinal changes in body weight during high-fat diet (HFD) administration. **a** Body weight of WT and TG2−/− mice at the starting point, 6-week-old mice, and after 16 weeks (22-week-old mice) of chow diet or HFD administration. **b** Body weights of WT and TG2−/− mice were measured weekly from the same individuals, over the 16 weeks of HFD experimental period. Body weight gain is expressed as a percentage of the initial body weight. Data are the mean ± SD (*n* = 10). *p < 0.05; **p < 0.005

**Fig. 2:**
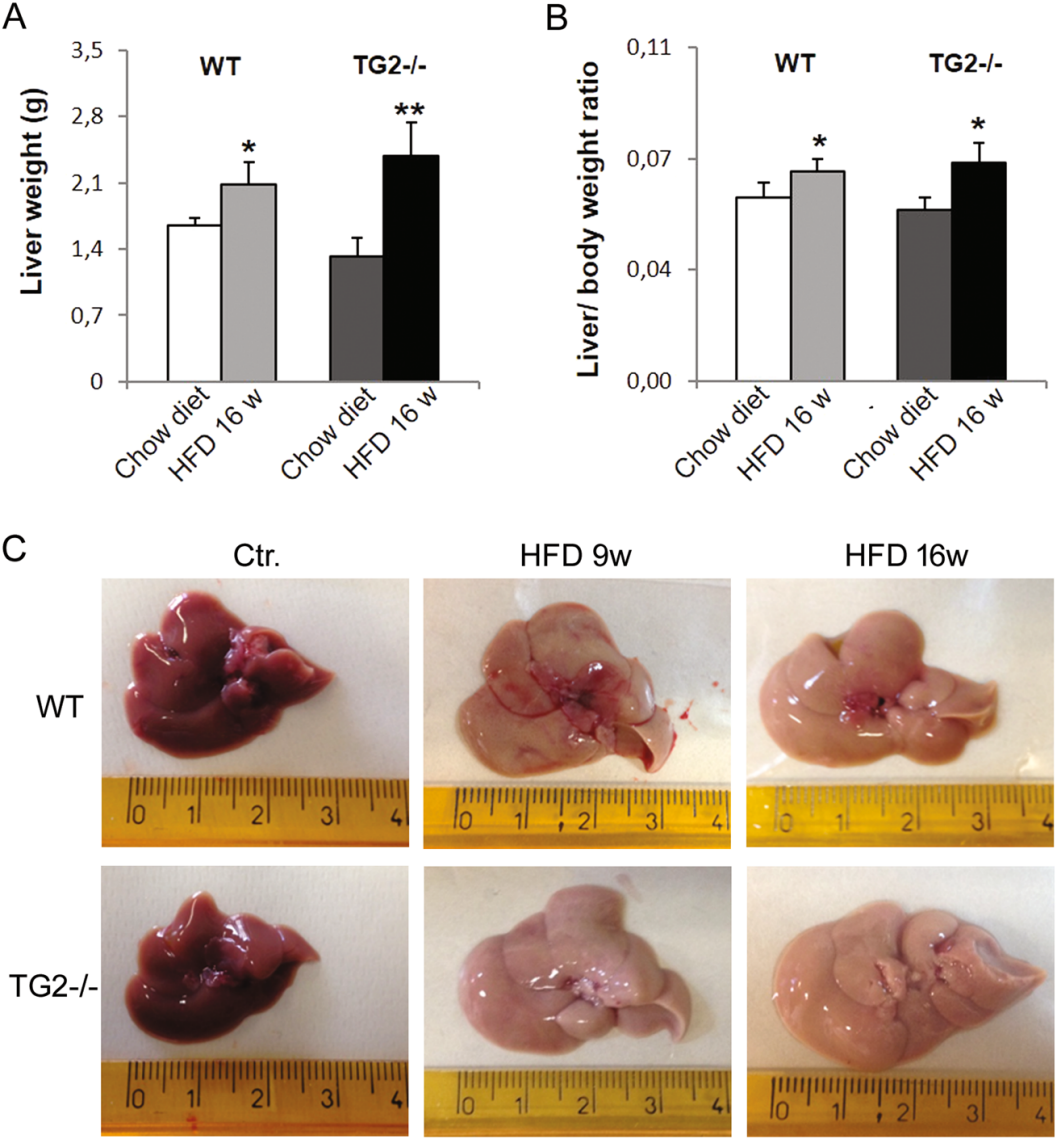
Liver macroscopic changes induced by HFD in WT and TG2−/− mice. **a** Graphical comparison of the gross liver weights after 16 weeks of feeding with chow diet or HFD: knockout mice showed a significant increase (80%) in the animals fed high-fat diet when compared with mice fed a regular diet. **b** Increase of liver weight to body weight ratio in the mice fed high-fat diet when compared with fed chow diet. **c** No differences are visible between control WT and TG2−/− mice in either coloration and gross appearance of the liver. Consumption of a high-fat diet resulted in an increase in gross liver size and yellowish coloration progressively over time (size WTD vs KOD *p* < 0.05). Ctr = normal chow-fed. Data are the mean ± SD (*n* = 10). *p < 0.05 and **p < 0.01, HFD vs Ctr

To investigate the impact of TG2 in NAFLD progression, wild-type (WT) and TG2−/− C57BL/6 mice were fed with high-fat diet (HFD) for 16 weeks, starting from 6 weeks of age, and the body weight increase was monitored once a week. At the the start of the diet the knockout (KO) mice had a slightly lower body weight compared to the WT (Fig. 1a), however at the end of the 16 weeks of the treatment they gained weight similar to WT mice (Fig. 1a). As expected, HFD feeding promoted obesity, with significant weight gain as compared to controls for both WT and TG2−/− animals. Interestingly, after 16 weeks of diet, WT mice gained 68% of their original body weight vs 120% in the case of KO mice (*p* < 0.005; Fig. 1b), suggesting that liver and adipose tissue could account for the differences. Indeed, liver weight gain increased significantly in KO mice and a significantly more pronounced increase in the ratio of liver to body weight was found in KO vs WT animals (Fig. 2b). Enhanced visceral fat mass was also observed in TG2−/− mice during dissection (Suppl Fig. 1).

### NAFLD development

**Fig. 3:**
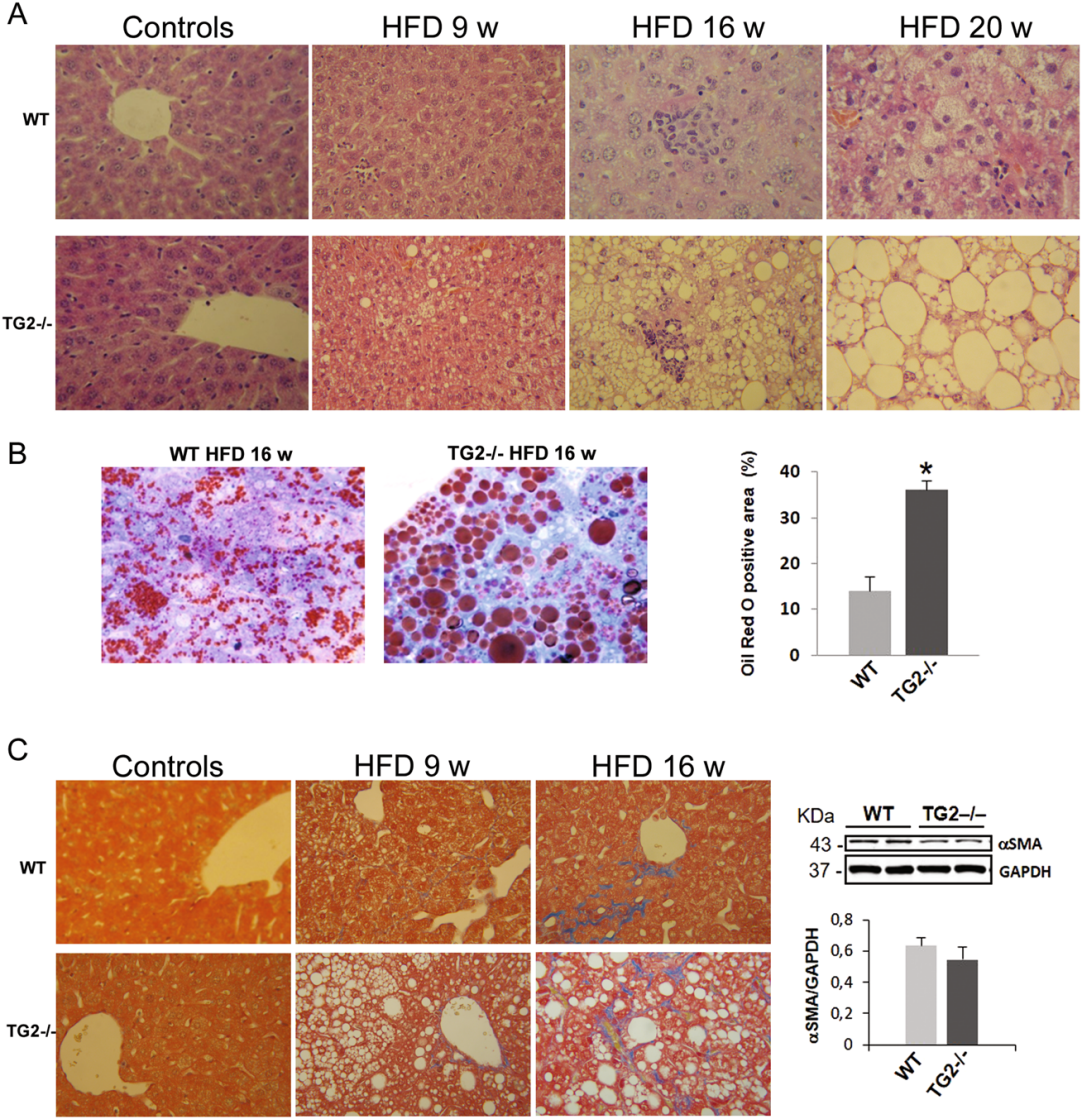
Histological alterations of the liver in HFD-fed WT and TG2−/− mice. **a** Representative hematoxylin–eosin-stained liver sections from normal chow-fed (Ctr) mice and from mice exposed to high-fat diet (HFD) for 9, 16, or 20 weeks. TG2−/− mice display severe hepatic steatosis (×10 and ×40 original magnification). **b** Representative Oil Red staining images depicting different pattern of lipid accumulation in HFD-fed WT and TG2−/− mice; on the right, quantification of lipid droplets (percentage of positive area) using Oil Red O staining. Data are the mean ± SD. *n* = 6 animals/group, *p < 0.05. **c** Masson’s trichrome staining of liver sections from HFD-fed mice reveals signs of perisinusoidal fibrosis (×10 and ×40 magnification); representative western blot analysis and densitometric evaluation of α-smooth muscle actin (α-SMA) in liver of HFD-fed WT and TG2−/− mice are shown on the right. The data were presented as mean ± SD. *n* = 6 animals/group

Chronic exposure to HFD caused an increase of hepatic lipid content, a clinical hallmark of NAFLD. Macroscopic appearance of livers from HFD animals displayed a size increase and a marked discoloration due to fat accumulation (Fig. 2c). Both these features were more pronounced in TG2−/− with respect to WT (liver size, WT vs TG2−/−, *p* < 0.05; Fig. 2c). Hematoxylin-and-eosin (H&E)-stained sections from mice fed with chow diet displayed normal liver histology, with hepatocyte cytoplasm devoid of lipid vesicles (Suppl Fig. 3); no differences were present between WT and TG2−/− mice (Fig. 3a). WT animals consuming HFD showed development of hepatic steatosis, mostly represented by microvesicular steatosis, as assessed by Oil Red O staining (Fig. 3b), whereas a marked more severe induction of NAFLD was found in KO animals, which exhibited diffuse presence of macrovescicular lipid droplets (LDs; Fig. 3a, b).

Hepatocyte vacuolation correlated with increasing time consuming the HFD (Fig. 3a); in addition inflammation and ballooning degeneration were observed, indicative of a progression from simple steatosis to steatohepatitis. However, visualization of Masson’s trichrome-stained liver sections demonstrated no great difference in the deposition of collagen fibers between HFD treated WT and TG2−/− mice (Fig. 3b). We next analyzed the activation of the profibrogenic marker, α-SMA and observed no statistically significant difference in Wt vs the TG2−/− mice (Fig. 3b).

**Fig. 4:**
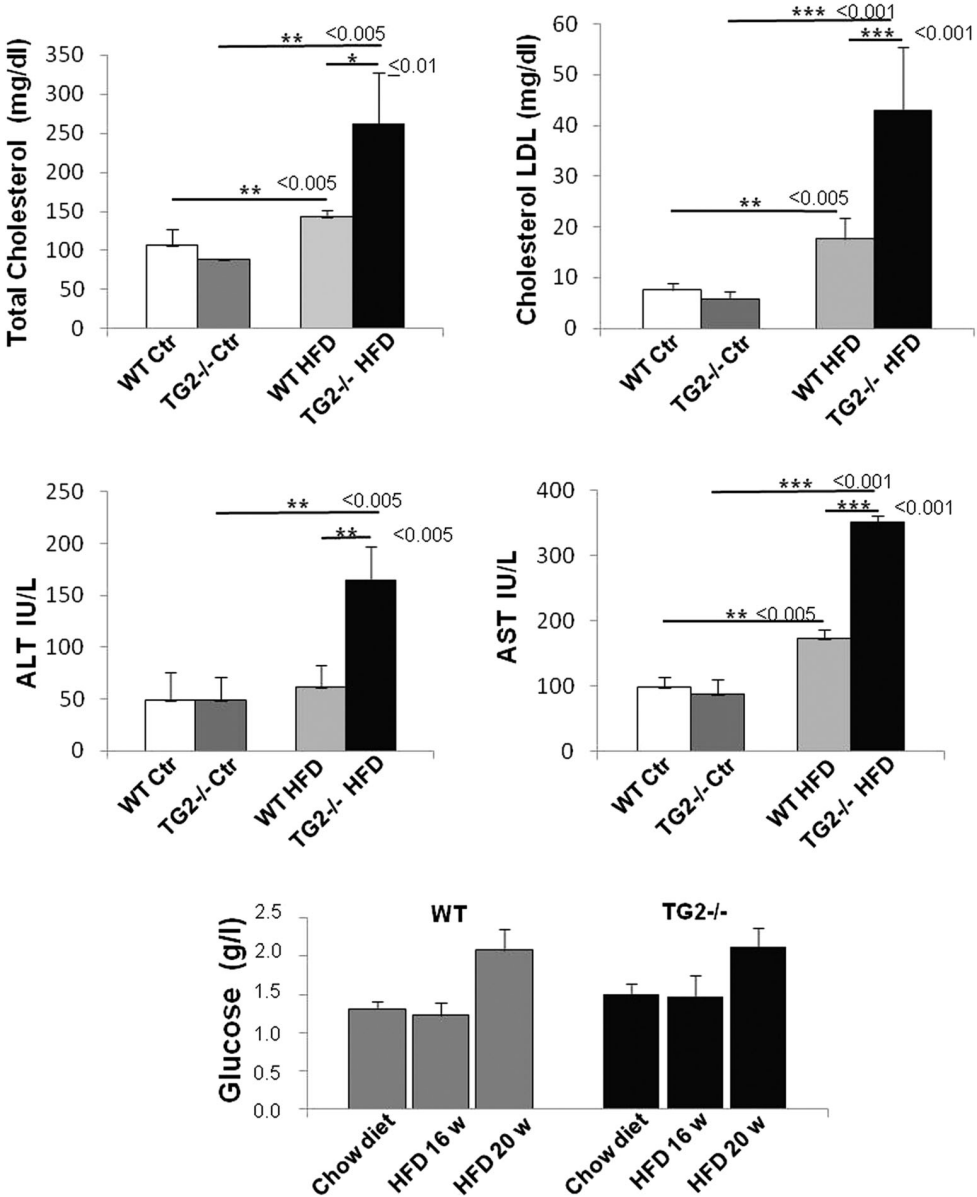
Analysis of biochemical parameters. Levels of total cholesterol, LDL-cholesterol, alanine aminotransferase (ALT) and aspartate aminotransferase (AST), and glucose in the serum of WT and TG2−/− mice receiving chow diet or HFD. All parameters appeared significantly increased by HFD consumption in the knockout mice, with respect to the controls and to the HFD WT, except for the glucose. A tendency to glucose increase was observed in both WT and TG2−/− mice HFD-fed for longer time. Each value is the mean ± SD (*n* = 10 animals/group). Significant differences are shown

To analyze the correlation between the progression of NAFLD and metabolic abnormalities, serum biochemical parameters were evaluated. Data obtained showed that TG2−/− mice HFD-fed for 16 weeks were hypercholesterolemic and display evidence of significant hepatic injury (blood alanine aminotransferase (ALT) and aspartate aminotransferase (AST); Fig. 4). HFD feeding induced increased values of total cholesterol (TC) and low-density lipoprotein (LDL)-cholesterol (LDL-C) in both mice strains, but in the KO mice these levels were significantly higher (TC: *p* ≤ 0.01; LDL-C: *p* ≤ 0.001; Fig. 4). In addition, in the absence of TG2, blood transaminases also significantly increased compared to WT (ALT: *p* ≤ 0.005; AST: *p* ≤ 0.001; Fig. 4), reaching a 3.4- and 4-fold increase with respect to control TG2−/− mice.

Treatment with HFD for 16 weeks did not induce hyperglycemia, either in WT or in TG2−/−mice. A tendency for an increase of blood glucose concentration was observed in mice in the 20-week diet, reaching values of about 1.4 times higher in both WT and TG2−/− compared to control mice (Fig. 4).

### Analysis of autophagy and mitochondrial homeostasis

**Fig. 5:**
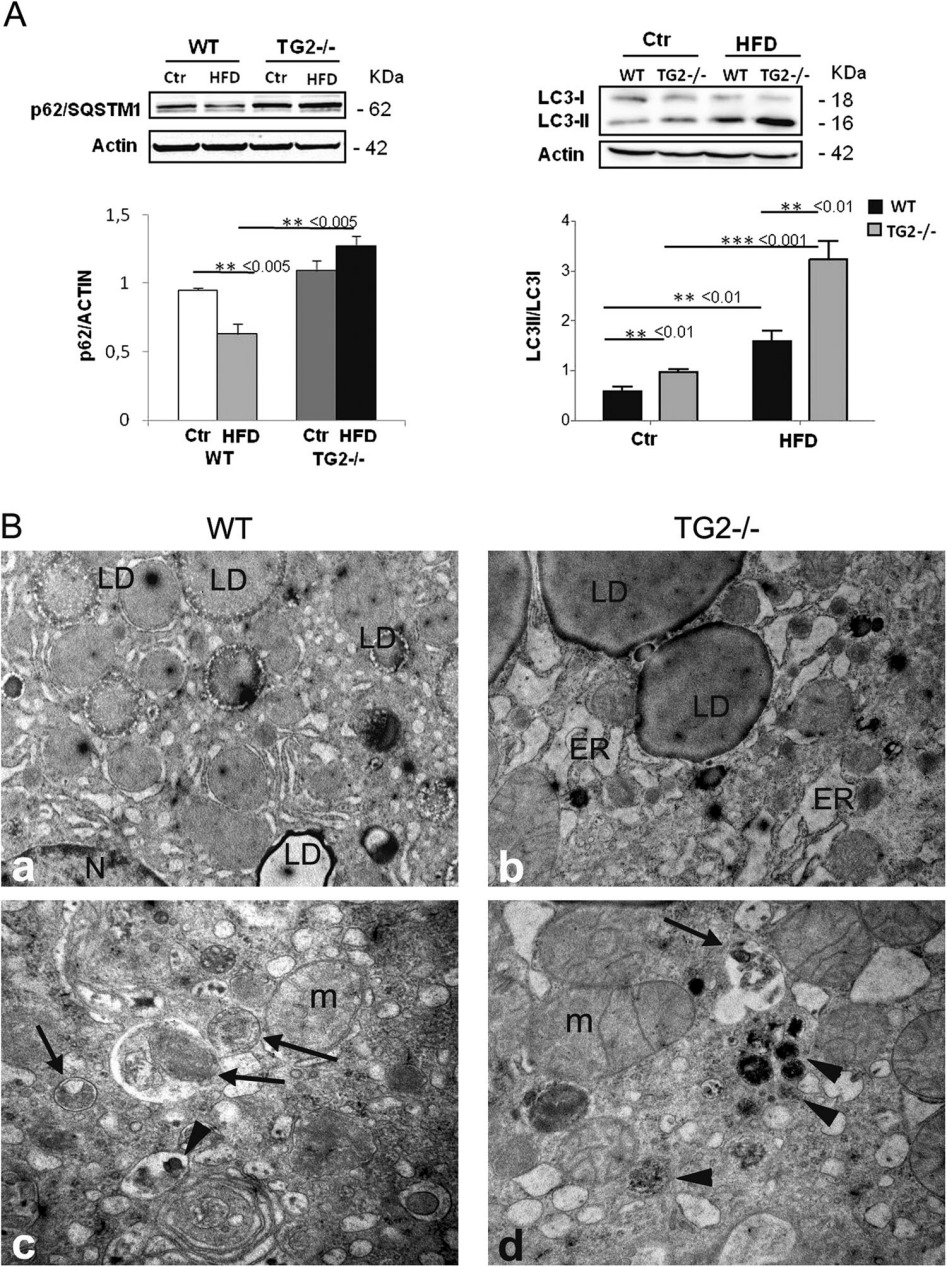
Alterations of autophagic process. **a** Representative western blot against p62/SQSTM1 and LC3II, in liver samples from WT and TG2−/− mice fed with chow diet (Ctr) or HFD for 16 weeks, anti-β-actin was used as an internal control. Quantification of p62/SQSTM1 band intensity relative to actin and the analysis of LC3II/LC3I ratio are shown, data were presented as mean ± SD. *n* = 6–10 animals/group. Significant differences are shown. **b** Transmission electron microscopy of liver from HFD-fed WT and TG2−/− mice. a, c Representative electron micrographs of WT liver: numerous lipid droplets (LDs) of varying density are visible (a); autophagosomes (arrows) and autolysosome with enclosed residual digested material (arrowhead) indicate intense autophagy induction (c) (N = nucleus). b, d Representative electron micrographs of TG2−/− liver: hepatocytes display very large LDs and massive endoplasmic reticulum (ER) dilatation (b); accumulation of vacuoles with partially digested material (arrows) and granular inclusions of protein aggregates (arrowhead) are visible. (Original magnifications: a, b = ×7000; c, d = ×20 000)

We demonstrated that the TG2 KO mice have an impairment of the autophagic process, which is particularly evident under stressful conditions^15^. On this basis we examined, by western blotting, LC3II and p62/SQSTM1 expression on liver samples from WT and TG2−/− mice fed HFD for 16 weeks. Although the analysis of LC3II/LC3I ratio shows LC3II conversion, after HFD diet, both in WT and TG2−/− mice, a higher LC3I to LC3II conversion was detected in TG2−/− mice (Fig. 5a), which was paralleled by the accumulation of p62/SQSTM1 (Fig. 5a) only in TG2−/− mice (Fig. 5a). Since p62/SQSTM1 functions as an adaptor protein between autophagic machinery and ubiquitinated proteins, it is constantly degraded by the autophagy–lysosome system, thus the decline of the molecule in HFD-fed WT mice is indicative of autophagic activity induction. By contrast, both the LC3II and p62/SQSTM1 accumulation confirm, also in this experimental setting, the reported impairment of the autophagic process in the TG2 KO mice. Transmission electron microscopy examination of livers from WT and TG2−/− mice HFD-fed for 16 weeks also confirmed the above-reported results. In fact, while hepatocytes from WT mice displayed autophagic vacuoles with enclosed organelles and autolysosomes with residual digested material (Fig. 5b (c)), the hepatocytes from HFD-fed TG2−/− mice showed accumulation of vacuoles with often poorly identified content, together with the presence of cytoplasmic inclusions of aggregated proteins (Fig. 5b (d)), further indicating impairment of the degradative autophagic pathway.

**Fig. 6:**
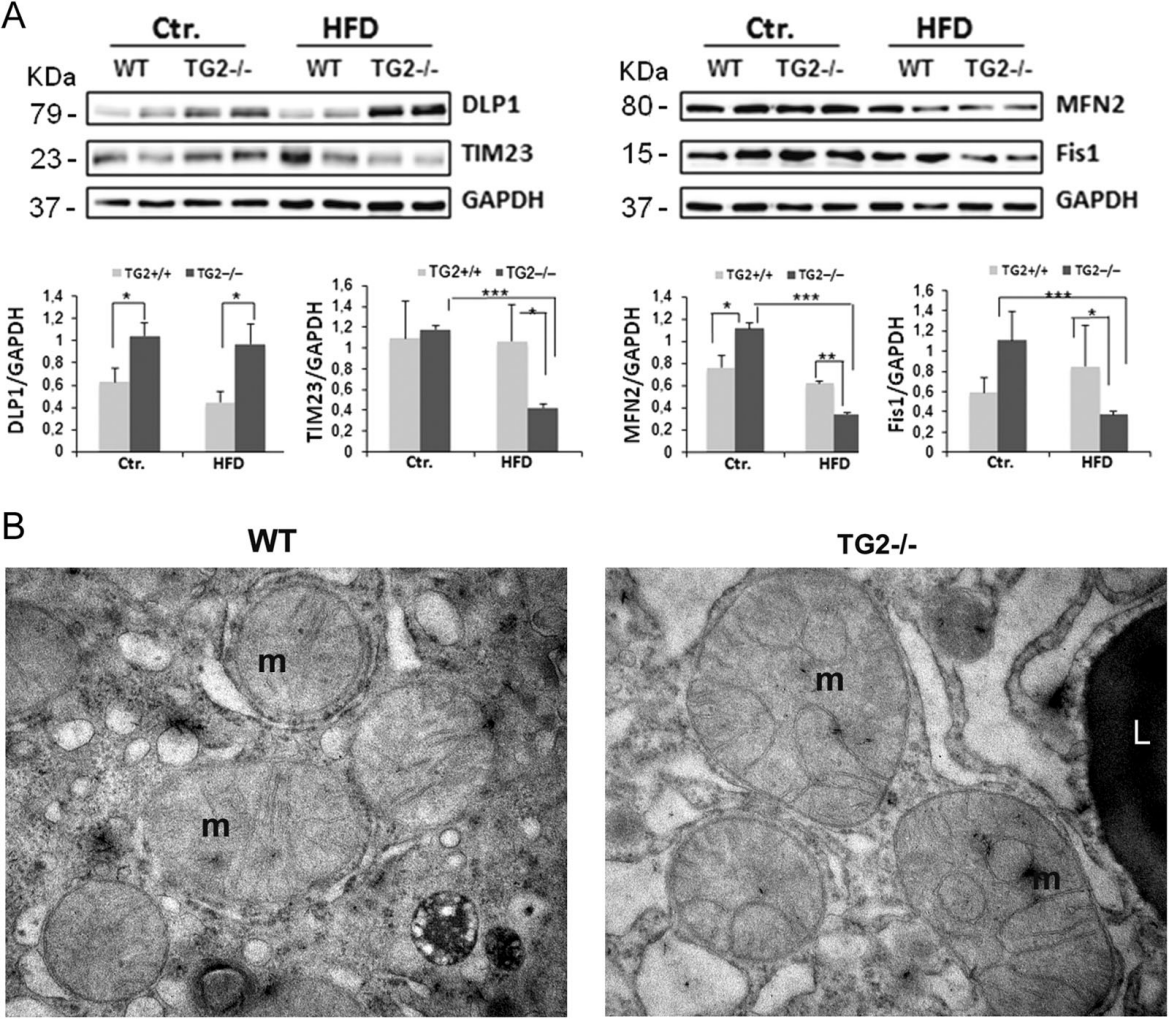
Analysis of mitochondrial dynamics. **a** Representative western blot against DLP1, Fis1, MFN2, and TIM23 in liver samples from WT and TG2−/− mice fed chow diet (Ctr) or HFD for 16 weeks (HFD). Quantification of band intensity relative to GAPDH is shown, data were presented as mean ± SD. *n* = 10 animals/group. *p < 0.05; **p < 0.01; ***p < 0.005. **b** Transmission electron microscopy of liver from HFD-fed WT and TG2−/− mice. In WT liver most mitochondria (m) show round profile and normal features; in TG2−/− liver mitochondria were heterogeneous in size and shape, and displayed abnormal cristae morphology. Original magnification: ×30 000

The ultrastructural analysis confirmed the different pattern of fat accumulation between WT and TG2−/− mice (Fig. 5b (a, b)), as well as changes in mitochondrial morphology. Considering that TG2 ablation leads to mitochondrial dysfunction^16^ and that the impairment of these organelles dynamics has been suggested to play a central role in the NAFLD development^17^ we analyzed the impact of HFD feeding on mitochondria homeostasis in the presence or absence of TG2. To this end we analyzed by expression level of key proteins involved in mitochondrial dynamics: the fusion mediator mitofusin-2 (MFN2), the fission mediator dynamin-like/related protein 1 (DLP1/DRP1), and the fission 1 protein (Fis1). Data obtained showed an increased level of Fis1 and a decrease of MFN2 protein amount in HFD-fed WT mice (Fig. 6a), suggesting the induction of the fission process. By contrast, the TG2 KO mice showed a drastic decrease in the Fis1 levels, associated with an increased DRP1 and a decrease MFN2 protein amount (Fig. 6a), indicating a general impairment of the mitochondria fission/fusion processes. We also evaluated the expression level of TIM23 to assess whether damaged mitochondria retained a functional import machinery. Of note HFD-fed TG2−/− mice displayed a strong decrease of TIM23 protein amount (Fig. 6a), again supporting the imbalance of mitochondria quality control mechanism in the absence of TG2. Electron microscopy observations showed that mitochondria of livers from HFD-fed WT mice mostly appeared to be uniformly round shaped (Fig. 6b), often being in contact, suggesting that division processes took place. Observation of hepatocytes from HFD-fed TG2−/− mice revealed the presence mitochondria often abnormally shaped, with unusual cristae morphology, which appear as infoldings of the inner membrane (Fig. 6b), again suggesting derangement of the fission/fusion dynamics.

### TG2 inhibition in WT animals

**Fig. 7:**
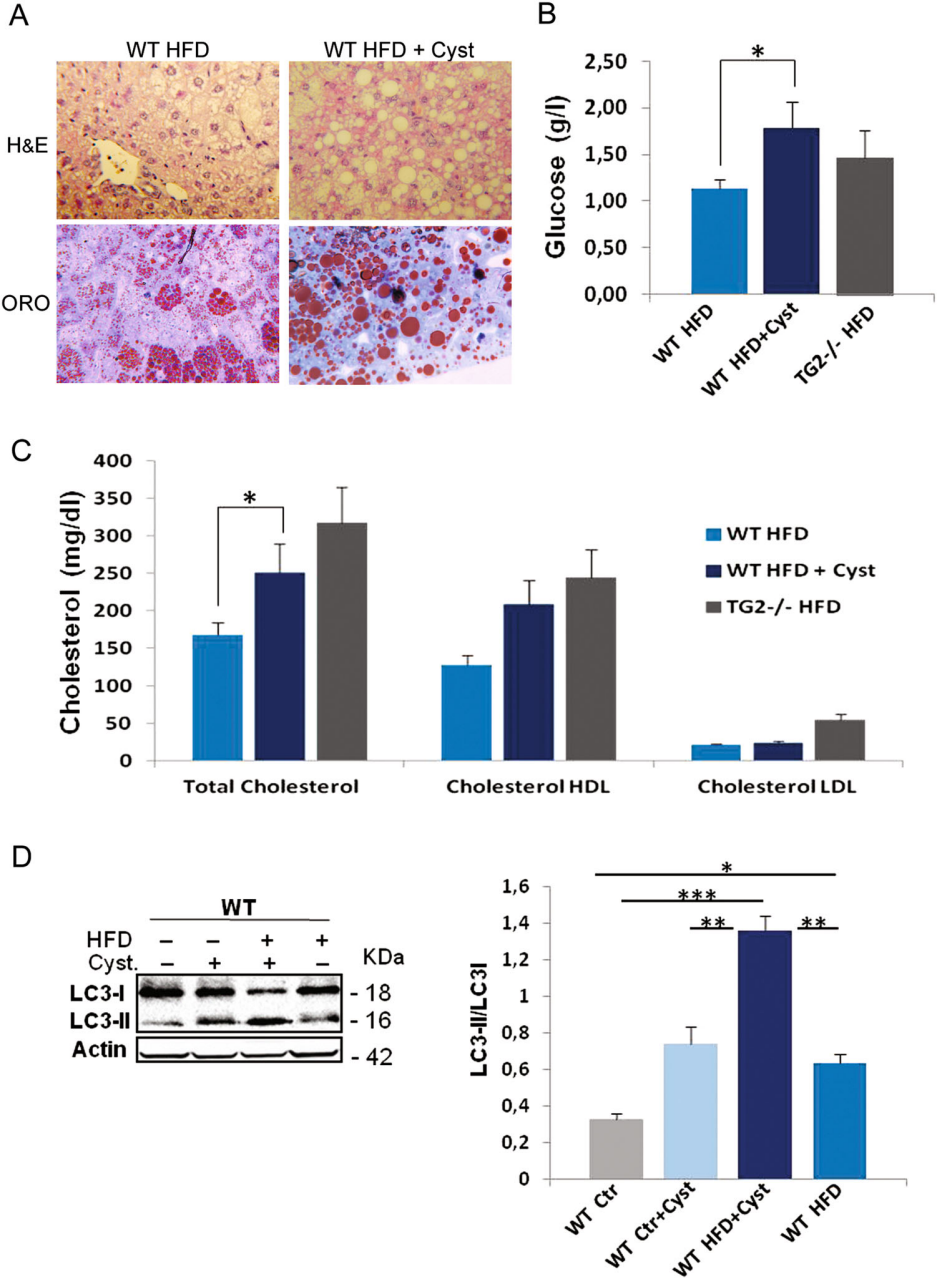
Effect of cystamine treatment in WT mice. HFD-fed WT mice were treated with cystamine (CYST) for 2 weeks before being sacrificed at week 16 of diet administration. **a** Representative hematoxylin–eosin (H&E) and Oil Red O (ORO)-stained liver sections display massive macrovesicular steatosis in liver of cystamine-treated animals (original magnification ×40); **b** blood glucose and **c** cholesterol levels in cystamine-treated WT mice showed a significant increase, resembling HFD-TG2−/− mice. **d** Left panel: representative western blot analysis against LC3II in liver samples from WT mice fed chow diet (Ctr) or HFD in the presence or absence of cystamine. Anti-β-actin was used as an internal control; right panel: analysis of LC3II/LC3I ratio. Data are presented as mean ± SD from five mice per group. *p < 0.05; **p < 0.01; ***p < 0.005

In a further group of HFD WT mice the TG2 transamidating activity was inhibited utilizing cystamine^18^. The drug was orally administered for 2 weeks, starting from the week 14 after the beginning of HFD regimen. Results obtained showed that cystamine administration exacerbates the effects of the HFD on liver fat accumulation, with macrovesicular steatosis becoming predominant over microvesicular steatosis (Fig. 7a), very similar to what was observed in the KO mice. After cystamine treatment glycemia and cholesterolemia values also proved to be significantly increased (Fig. 7b, c). It is important to note that when the WT animal fed with HFD were treated with TG2 inhibitor, a significantly higher level of LC3II was also detected in the liver extracts (Fig. 7d).

### TG2 expression in human liver from NAFLD patients

**Fig. 8:**
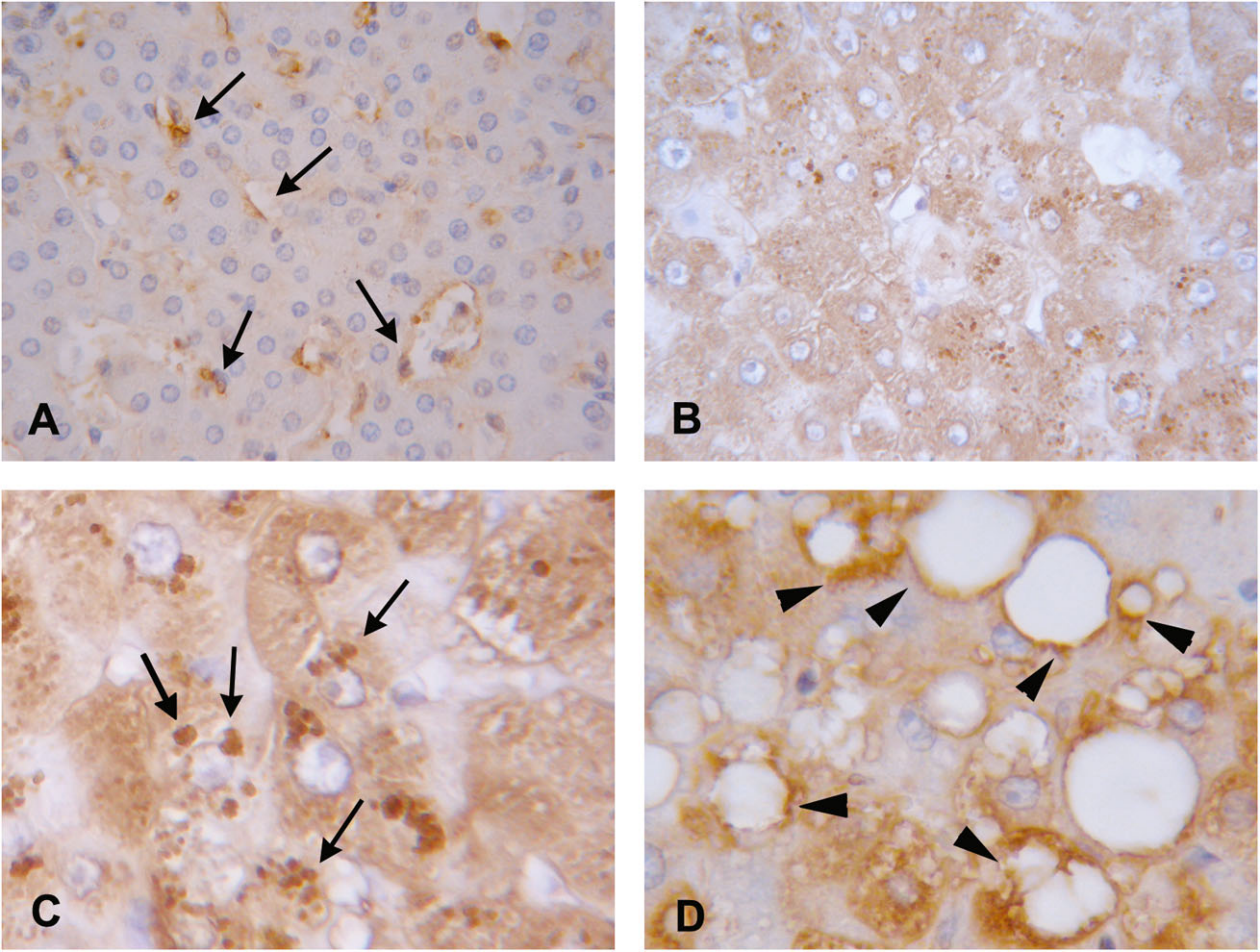
TG2 expression in human liver from NAFLD patients. Representative immunohistochemical features of TG2 staining in normal (**a**) and non-alcoholic fatty liver disease (**b**–**d**). **a** In normal liver the staining is visible in endothelial cells of the sinusoids (arrows). **b** NAFLD liver shows hepatocyte positivity: **c** higher magnification of a microvesicular steatosis area characterized by puncta distribution of the staining (arrows); **d** TG2 labeling around lipid droplets (arrowheads) in an area of macrovesicular steatosis. Original magnification: **a**, **b** ×40; **c**, **d** ×63

To explore whether fat accumulation in human liver could determine changes of expression of TG2, an immunohistochemical analysis was performed on liver samples from patients affected by NAFLD. In normal liver, immunostaining resulted in undetectable TG2 in hepatocytes, while a positive reaction was observed in sinusoidal endothelial cells (Fig. 8a). Interestingly, parenchymal cells exhibited an intense TG2 labeling in the steatotic liver (Fig. 8b–d). Of note, in areas affected by microsteatosis the staining was found as puncta in the cytoplasm (Fig. 8b, c), whereas in areas with macrosteatosis the staining appeared distributed around or in proximity of lipid vesicles (Fig. 8d). This different expression confirmed the involvement of TG2 during steatosis development.

## Discussion

NAFLD is probably the most common liver disorder in the world. The spectrum of NAFLD ranges from simple steatosis to NASH, liver fibrosis, cirrhosis, and hepatocellular carcinoma^4^. Even though several mechanisms have been proposed to explain the molecular basis for the fat accumulation, liver injury, and fibrosis, still many gaps exist in the understanding of the NAFLD pathogenesis^5,19^.

Results obtained in this study showed that the lack of TG2 elicits the worsening of the disease progression, caused by a greater accumulation of lipid macrovesicles. The severity of the disease was confirmed by the serum biochemical analyses that showed a higher blood concentration of cholesterol and transaminases in TG2−/− compared to WT mice.

We have previously shown that the absence of TG2 leads to the impairment of autophagy/mitophagy^15,16,20^ and that very likely this represents the main mechanism responsible for the worsening of NAFLD development observed in the TG2 KO animals. In fact, much evidence indicates that autophagy plays a crucial role in the regulation of lipid metabolism^21^ and that autophagy deficiency increases intracellular fat accumulation, whereas autophagy induction improves hepatic steatosis^22,23^. In agreement with our previously published findings, TG2−/− mice fed with HFD showed the accumulation of the autophagic markers, p62 and LC3II, indicating a block of the autophagic process. Considering that in the absence of TG2 the block of autophagy is particularly marked under stressful conditions, during a long-term lipid load condition the lack of the enzyme could further exacerbate autophagy impairment and accelerate lipid accumulation.

Aberrant activation of TG2 or deregulation of its function(s) is involved in a variety of human diseases, such as celiac disease, diabetes, neurodegenerative diseases, inflammatory disorders, and septic shock^24–26^. In our experimental model, no significant increase of the protein was found in the liver of WT animals fed with HFD (Suppl Fig. 2). However, the biological function of TG2 is often not regulated by changes in its protein amount but is related to conformational changes due to environmental modification such as the balance between GTP vs Ca++ levels^27^. Indeed, in injured liver, there is an increased intracellular Ca^++^ elevation^28,29^. In addition, even changes of TG2 localization inside the cell have been shown to be related to activation of specific functions of the enzyme^11^. Interestingly, we observed a change in TG2 distribution in NAFLD human liver, with a specific localization restricted to particular cell compartments.

Dysregulation of different factors acting in parallel has been proposed to explain the development of NAFLD. Evidence suggests a role for reactive oxygen species (ROS) in progression of NASH^30^. Under stress conditions, the oxidatively damaged proteins are prone to form tangled aggregates unless they are degraded by the ubiquitin-proteasome/autophagosomes system in a timely manner^31,32^. We have previously shown that the TG2 transamidating activity favors the formation of intracellular ubiquitinated protein aggregates, which are recognized by cargo proteins, such as p62 and NBR1, leading to their recruitment in the pre-autophagic vesicles before degradation by autophagolysosomes^20^. Thus, we hypothesize that the presence of TG2 exerts a beneficial rather than negative effect on NAFLD progression playing an important protective role. In line with this point of view are recently published data concerning the protective role of the formation of large insoluble aggregates against the cellular oxidative stress^33^. Intriguingly, the TG2 crosslinking activity plays a key role in the formation of Mallory-Denk bodies (MDBs), which are protein aggregates consisting of ubiquitinated keratins characteristic of alcoholic and NASH^34^. The TG2-dependent formation of highly crosslinked MDBs is functional to their elimination via macroautophagy^20^. In the absence of the enzyme, the misfolded keratins and other proteins that should be assembled into these aggregates, might lead to uncontrolled damaging effects on the hepatocytes^35^. Continuous oxidative stress and ROS formation has also been reported to encourage hepatocytes to enhance their lipogenic activity and to form ectopic LDs in greater numbers^36^. This fact may help to explain why the TG2−/− mice have faster progression to macrovescicular steatosis with dramatic expansion of LD size, compared to WT mice.

An additional way by which the impairment of TG2 can exacerbate fat deposition in the liver is related to the mitochondria homeostasis. In fact, mitochondrial dysfunction is a major and common mechanism in developing NAFLD^37^. In this study we show that after HFD treatment, mitochondria of TG2 KO mice displayed a clear impairment of their dynamics. These data are in line with our previous studies showing that mitochondria from TG2−/− mice display decreased membrane potential, downregulation of IF1 along with increased DRP1 and PINK1 levels (two key proteins regulating the mitochondrial fission). Thus, the absence of TG2 also leads to a mitochondrial homeostasis impairment that contributes to the development of the liver failure.

In line with the herein-reported role of TG2 in NAFLD development, of interest is the fact that Celiac disease (CD) patients seem to be at increased risk of NAFLD compared to the general population^38^. Since the presence of autoantibodies against TG2 is the hallmark of the CD patients, one can speculate that those antibodies might inhibit TG2 enzymatic activity, causing a greater propensity of these patients to NAFLD development. In keeping with this hypothesis is the effect of the TG2 inhibitor cystamine on a group of WT mice fed with a HFD. In fact, the data obtained again confirmed a substantial worsening of liver steatosis and liver injury in the absence of a functional TG2.

In conclusion, our data demonstrate that TG2 by controlling autophagy and mitochondrial homeostasis might play a protective role on the progression of NAFLD (Fig. 6). This fact is also suggested by the observed change in TG2 localization in human steatotic liver, which could represent a response of injured tissue. In the future, it will be interesting to study patients with different severity of the progression of NAFLD and analyze if there is any connection with a deficit or malfunction of the TG2. If confirmed this hypothesis could lead to the discovery of a new therapeutic target for this pathology.

## Materials and methods

### Animal experiments

Guidelines for Care and Use of Laboratory Animals were followed during the investigation. All animal procedures were approved by the Ethical Commission of the University of Rome “Tor Vergata” and performed according the principles established by the National Institutes of Health and by the Italian Ministry of Health. C57BL/6 mice, WT and TG2 KO (TG2−/−)^39^ were used. Animals were housed individually in an environmentally controlled room (18–21 °C, 40–70% relative humidity) with ad libitum access to water and food and kept under a 12-h dark/12-h light cycle.

### Experimental protocol

Male mice were used for all experiments. Animals were 6 weeks of age at the start of the study. WT male C57BL/6 mice were maintained on either a standard laboratory chow (Ctr) diet, or a “HFD/western-style” diet (*n* = 10/group) with 42% kcal from fat (HF, TD.88137, Harlan Teklad (North America)) for 16 weeks. Body weights were monitored once weekly throughout the experiments.

In a further group, the effects of cystamine, a TG2 inhibitor, were studied. Cystamine (Sigma-Aldrich; St. Louis, MO, USA) was delivered orally in drinking water. Based on water consumption of 5 ml/day/20 gm mouse, a cystamine concentration of 900 mg/l tap water was used to deliver an approximate dose of 225 mg/kg^40^. Fresh cystamine solution was made weekly. Ctr or HFD-fed WT mice (*n* = 5/group) were treated with cystamine for a period of 2 weeks before being killed.

### Dietary features of HFD

Cholesterol (0.2% total cholesterol); total fat (21% by weight; 42% kcal from fat); high in saturated fatty acids (>60% of total fatty acids); and high sucrose (34% by weight).

### Sample collection

Blood samples were collected from the retro-orbital sinus and centrifuged to obtain serum; samples were stored at −80 °C for further analysis. At the end of experiments, mice were killed by cervical dislocation. Thoracic and abdominal organs were examined macroscopically. The liver from each mouse was rapidly removed and weighed, and then divided into portions for histological and ultrastructural examination, and for immunoblotting analysis.

### Histopathological analysis

Liver tissues were fixed in 10 % neutral-buffered formalin, and paraffin-embedded. Sections of liver (4 μm) were stained with H&E and blindly evaluated for assessment of steatosis and steatohepatitis. Hepatic fibrosis was analyzed by Masson-trichrome staining for collagen fibers. The accumulation of LDs was examined by Oil Red O staining. Semithin sections of Epon-embedded samples were stained in pre-warmed Oil Red O solution (Sigma-Aldrich) at 60 °C. Stained slides were counterstained with with methylene blue. Ten fields/liver section per each group were digitally imaged for analysis (×40 objective). Quantification of LDs (percentage of Oil Red O-stained area) was measured with the ImageJ (version 1.44C) software.

### Transmission electron ultrastructural analysis

Tissue samples were fixed with 2.5% glutaraldehyde (Assing Spa, R1012) in 0.1 M cacodylate buffer for 1 h at 4 °C (sodium cacodylate trihydrate, Sigma-Aldrich, C4945), and postfixed in 1% osmium tetroxide (Sigma-Aldrich, 75632) in 0.1 M cacodylate buffer for 1 h. Samples were then dehydrated in graded ethanol and embedded in Epon resin (AGAR 100, Agar Scientific R1045). For ultrastructural analysis thin sections were stained with 2% uranyl acetate and observed under a Zeiss EM900 transmission electron microscope. Images were captured digitally with a Mega View II digital camera (SIS, Soft Imaging System GmbH, Munster, Germany).

### Blood biochemistry

Collected sera were sent to the blood profiling service of Spallanzani Clinical Laboratory for measuring ALT, AST, triglycerides, glucose, total cholesterol, LDL-cholesterol, and HDL-cholesterol concentration.

### Western blot analysis

Livers were homogenized in 50 mM Tris HCl (pH 7.5), 50 mM NaCl, 320 mM sucrose, 1% Triton X-100, and 10% glycerol supplemented with protease and phosphatase inhibitor cocktail. Protein concentrations were determined by the Bradford assay, using bovine serum albumin as a standard. Aliquots of total protein extracts from cells and tissues after different treatments were resolved on SDS-polyacrylamide gel and transferred to a nitrocellulose membrane. Blots were blocked in 5% non-fat dry milk in T-PBS (phosphate-buffered saline (PBS) + 0.05% Tween 20) for 1 h at room temperature and then incubated overnight with the following primary antibodies: anti-TG2 (Cell Signaling); anti-p62/SQSTM1 (mbl, PM045); anti-LC3 (Novus Biologicals, NB100-2331); anti-GAPDH (Sigma, G9545); and anti-Actin (Sigma, 2066). The membranes were incubated with horseradish peroxidase-conjugated secondary antibody for 1 h at room temperature and the signal was detected by Immun-Star WesternC Kit (Bio-Rad Laboratories).

### Human samples

Sections of human liver were obtained from formalin-fixed and paraffin-embedded blocks of retrospective/archival samples (i.e., patient selection and liver biopsy were not for the purpose of the study). Replicate blocks, no longer required to be maintained on file within the local pathology archive, were used. Six cases of NAFLD and six cases of normal control tissue were selected; the presence of a hepatitis B or hepatitis C infection, or of alcoholic liver disease were considered as exclusion criteria.

For immunohistochemistry, deparaffinized and rehydrated sections were immersed in 10 mM sodium citrate, pH 6.0, and microwaved for antigen retrieval. Samples were incubated with mouse anti-TG2 (CUB 7402, Thermo Scientific, Rockford, IL, USA), for 1 h at room temperature. Reaction was visualized using a streptavidin–biotin–immunoperoxidase system with DAB (Biogenex, San Ramon, CA) as chromogen substrates. Negative control staining was performed by omitting the primary antibody. Sections were counterstained in Mayer’s acid hemalum.

### Statistical analysis

Statistical significance between normal chow group and HFD group and between WT and KO mice was analyzed by two-sided, unpaired Student *t*-test. *p*value <0.05 was considered statistically significant. Data were represented as mean ± standard deviation.

## Electronic supplementary material

Supplemental Figure 1

Supplemental Figure 2

Supplemental Figure 3
