# Supplementary figures and images for: Non-alcoholic fatty liver disease severity is modulated by transglutaminase type 2
Source: Cell Death Dis. 2018 Feb 15;9(3):257. doi: 10.1038/s41419-018-0292-8 (PMC5833377; doi:10.1038/s41419-018-0292-8)

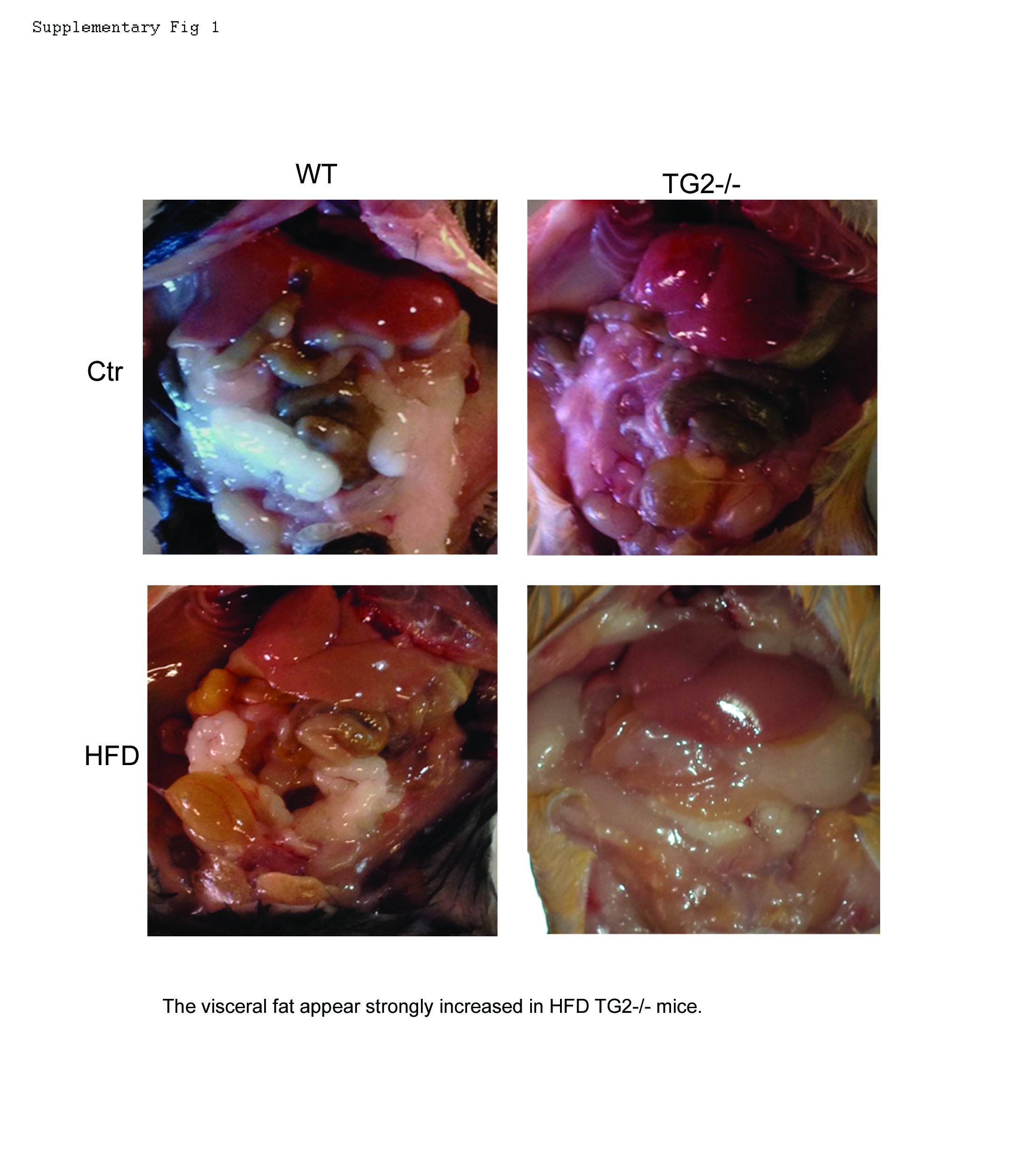

Supplement: Supplementary file 1 — Supplemental Figure 1 [file 41419_2018_292_MOESM1_ESM.jpg]

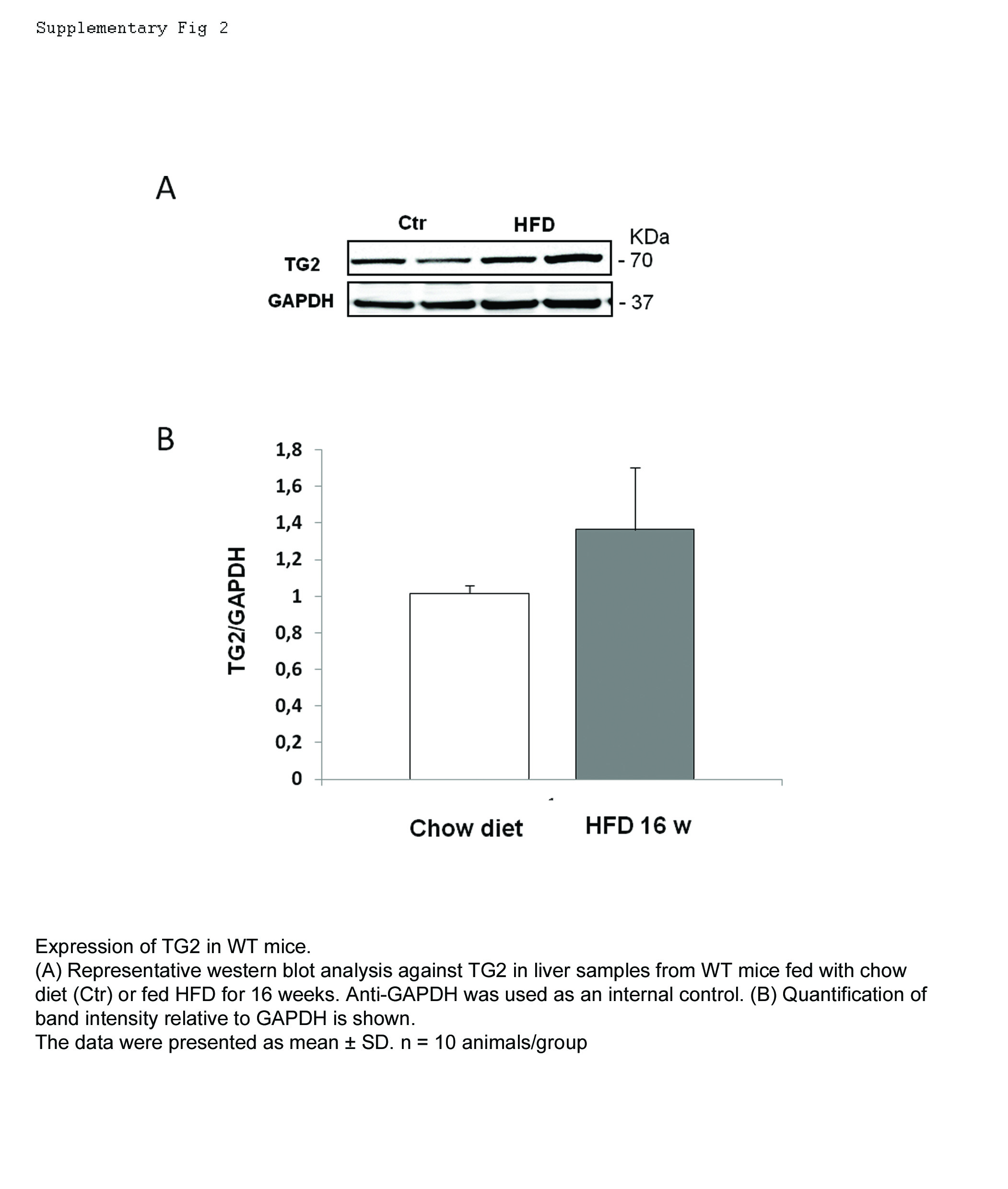

Supplement: Supplementary file 2 — Supplemental Figure 2 [file 41419_2018_292_MOESM2_ESM.jpg]

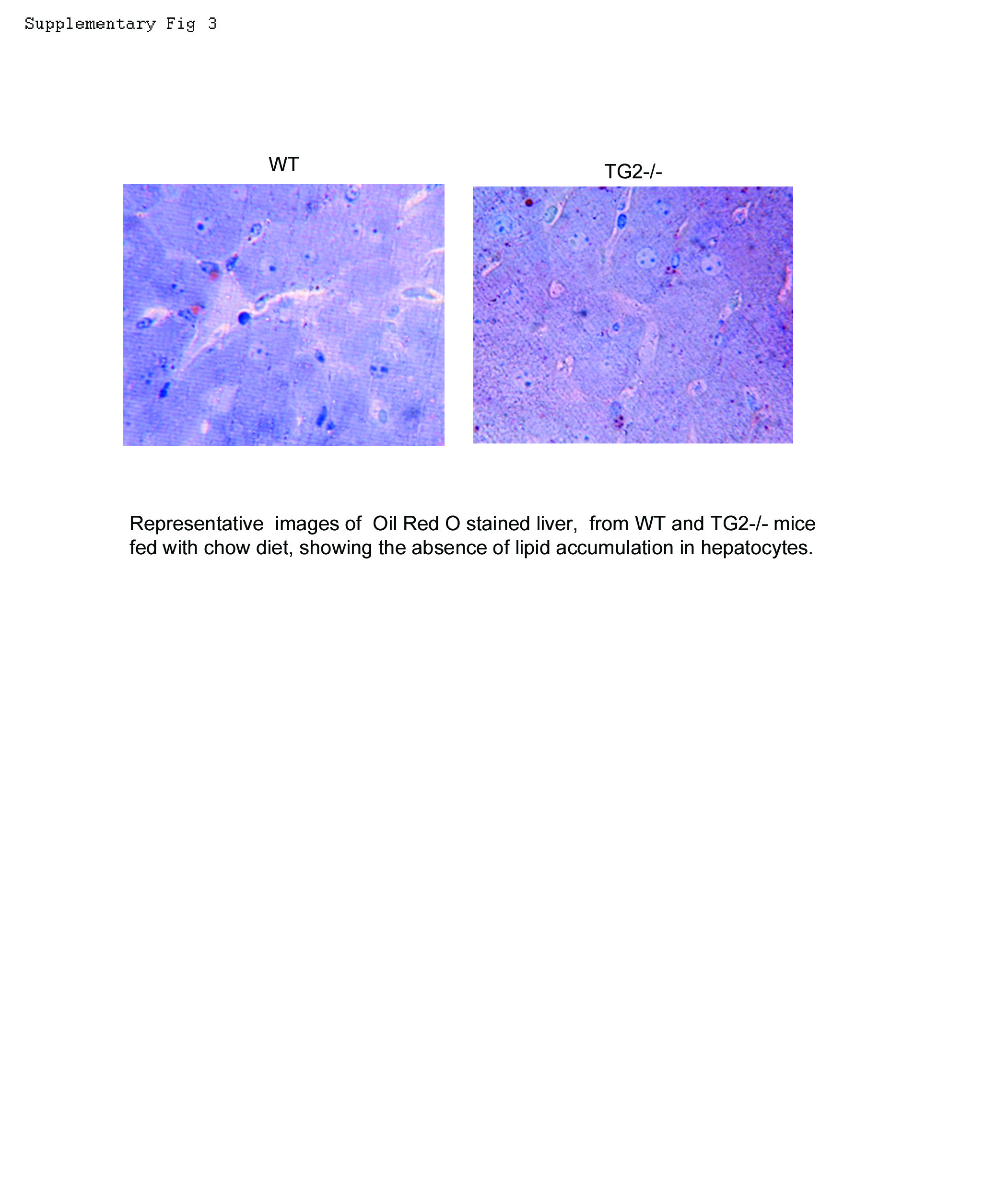

Supplement: Supplementary file 3 — Supplemental Figure 3 [file 41419_2018_292_MOESM3_ESM.jpg]
